# Activation of adenosine A_2B _receptors enhances ciliary beat frequency in mouse lateral ventricle ependymal cells

**DOI:** 10.1186/1743-8454-6-15

**Published:** 2009-11-18

**Authors:** Jonathan R Genzen, Dan Yang, Katya Ravid, Angelique Bordey

**Affiliations:** 1 Department of Laboratory Medicine, Yale University School of Medicine, New Haven, CT 06520-8082, USA; 2 Departments of Neurosurgery & Cellular and Molecular Physiology, Yale University School of Medicine, New Haven, CT 06520-8082, USA; 3 Departments of Biochemistry, Medicine, and Whitaker Cardiovascular Institute, Boston University School of Medicine, Boston, MA 02118, USA; 4 Department of Pathology and Laboratory Medicine, Weill Cornell Medical College, New York-Presbyterian Hospital, New York, NY 10065, USA

## Abstract

**Background:**

Ependymal cells form a protective monolayer between the brain parenchyma and cerebrospinal fluid (CSF). They possess motile cilia important for directing the flow of CSF through the ventricular system. While ciliary beat frequency in airway epithelia has been extensively studied, fewer reports have looked at the mechanisms involved in regulating ciliary beat frequency in ependyma. Prior studies have demonstrated that ependymal cells express at least one purinergic receptor (P2X_7_). An understanding of the full range of purinergic receptors expressed by ependymal cells, however, is not yet complete. The objective of this study was to identify purinergic receptors which may be involved in regulating ciliary beat frequency in lateral ventricle ependymal cells.

**Methods:**

High-speed video analysis of ciliary movement in the presence and absence of purinergic agents was performed using differential interference contrast microscopy in slices of mouse brain (total number of animals = 67). Receptor identification by this pharmacological approach was corroborated by immunocytochemistry, calcium imaging experiments, and the use of two separate lines of knockout mice.

**Results:**

Ciliary beat frequency was enhanced by application of a commonly used P2X_7 _agonist. Subsequent experiments, however, demonstrated that this enhancement was observed in both P2X_7_^+/+ ^and P2X_7_^-/- ^mice and was reduced by pre-incubation with an ecto-5'-nucleotidase inhibitor. This suggested that enhancement was primarily due to a metabolic breakdown product acting on another purinergic receptor subtype. Further studies revealed that ciliary beat frequency enhancement was also induced by adenosine receptor agonists, and pharmacological studies revealed that ciliary beat frequency enhancement was primarily due to A_2B _receptor activation. A_2B _expression by ependymal cells was subsequently confirmed using A_2B_^-/-^/β-galactosidase reporter gene knock-in mice.

**Conclusion:**

This study demonstrates that A_2B _receptor activation enhances ciliary beat frequency in lateral ventricle ependymal cells. Ependymal cell ciliary beat frequency regulation may play an important role in cerebral fluid balance and cerebrospinal fluid dynamics.

## Background

The cerebral ventricles are lined by a layer of ciliated ependymal cells that play an important role in cerebral fluid balance [1]. It has been estimated that each ependymal cell possesses 20-30 motile cilia, which are 8-20 μm in length with a 9 + 2 microtubule structure. Their ciliary tufts are organized in a manner consistent with the direction of cerebrospinal fluid (CSF) flow [2]. Abnormalities in ciliogenesis or ciliary function are frequently associated with hydrocephalus [3-11], and ependymal denudation can be observed in cases of communicating hydrocephalus [12]. Despite the increased recognition that ependymal cells are important for regulating CSF dynamics, only a few reports have specifically looked at the extracellular signaling mechanisms involved ependymal cell ciliary beat frequency modulation.

Nelson and Wright (1974) noted enhancement of frog brain ependymal ciliary beat frequency by ATP (adenosine 5'-triphosphate), cAMP (adenosine 3',5'-cyclic monophosphate), theophylline, and acetylcholine, as well as decreases in ciliary beat frequency by a number of other agents, using an *in vitro *preparation [13]. A later study by Nguyen *et al*. (2001) observed an ATP-mediated decrease in ciliary beat frequency, as well as a serotonin-mediated increase, in 4^th ^ventricle ependymal cells in cultured rat brain slices and acutely isolated ependymal cells [14]. Finally, reports from O'Callaghan *et al*. have demonstrated that both hydrogen peroxide and bacterial pneumolysin inhibit ciliary beat frequency in rat brain ependymal cells [15,16].

Recent work from our laboratory demonstrated that the purinergic P2X_7 _receptor is widely expressed on ependymal cells [17]. Furthermore, receptor activation leads to increases in intracellular calcium ([Ca^2+^]_i_) both in the soma and cilia. Working under the hypothesis that the P2X_7 _receptor may be involved in regulating ciliary beat frequency, we have conducted experiments using high-speed video capture and differential interference contrast (DIC) microscopy to investigate potential modulation of ciliary beat frequency by purinergic agonists. These experiments have demonstrated, however, that the adenosine A_2B _receptor is primarily responsible for ciliary beat frequency enhancement by these agents. Further experiments using A_2B_^-/-^/β-galactosidase reporter gene knock-in mice confirmed this observation and also demonstrated a residual P2X_7_-mediated component to ciliary beat frequency enhancement.

## Methods

### Slice preparation

**Figure 1:**
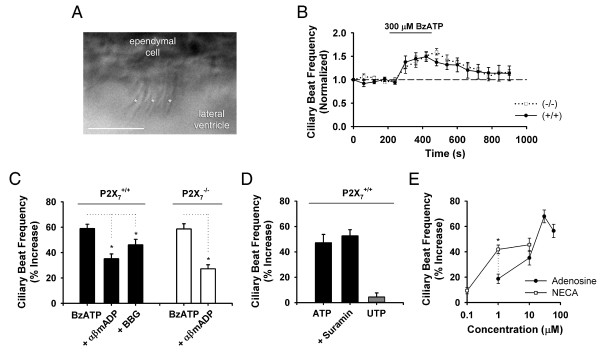
**Purinergic enhancement of ciliary beat frequency is observed in both P2X_7_^+/+ ^and P2X_7_^-/- ^mice**. (*A*) DIC image of an ependymal cell. (Bar = 12 μm) Cilia are noted (*). (*B*) Beat frequency (normalized) during 300 μM BzATP application in slices from P2X_7_^+/+ ^(filled circles/solid line; n = 5) and P2X_7_^-/- ^(open squares/dotted line; n = 4) mice. Responses from P2X_7_^+/+ ^and P2X_7_^-/- ^animals were not significantly different. (*C*) Beat frequency (% increase) in slices from P2X_7_^+/+ ^(n = 5) and P2X_7_^-/- ^(n = 4) mice after 5 min 300 μM BzATP application. Decreases (*P *< 0.05) were observed in the presence of the ecto-5'-nucleotidase inhibitor αβmADP (50 μM) in P2X_7_^+/+ ^(n = 6) and P2X_7_^-/- ^(n = 6) mice. BBG significantly decreased BzATP-induced enhancement in P2X_7_^+/+ ^mice (n = 4). (*D*) Beat frequency (% increase) in P2X_7_^+/+ ^mice during application of 100 μM ATP (*P *< 0.05 compared to no drug control; n = 4). The P2Y receptor antagonist suramin (200 μM; n = 5) did not significantly reduce enhancement seen with 100 μM ATP. Beat frequency was not enhanced by the P2Y_2_/P2Y_4 _agonist UTP (100 μM; n = 2). (*E*) Beat frequency enhancement with increasing concentrations of adenosine (filled circles; n = 4-5) and NECA (open squares; n = 4-8). A significant difference was observed between NECA and adenosine at 1 μM. *: *P *< 0.05 for all panels, data are means ± SEM. The *n *value indicates number of slices tested.

Research protocols were approved by the Yale University Institutional Animal Care and Use Committee (approval #A3230-01). C57BL/6 mice (n = 48; Jackson Laboratories, Bar Harbor, ME, USA), CD1 mice (n = 7; Charles River Laboratories, Wilmington, MA, USA), P2X_7 _knockout mice (n = 5; *P2rx7*^*tm1Gab*^, Jackson Laboratories, [18]), and A_2B _knockout mice (n = 7, [19]) were used for the present experiments. Mean age of animals was 24.3 ± 1.0 days (range 13-39). Animals were anesthetized with pentobarbital, 50 mg/kg, intraperitoneal (IP); after craniotomy and dissection, horizontal brain slices (250-300 μm) were prepared in chilled (4°C) dissection solution (in mM): 83 NaCl, 73 sucrose, 2.5 KCl, 2.7 MgCl_2_, 1.7 CaCl_2_, 1.2 NaH_2_PO_4_, 10 glucose, 26 NaHCO_3_, pH 7.4 and bubbled with 95% O_2_/5% CO_2 _using a series 1000 Vibratome (The Vibratome Company, St. Louis, MO, USA). Slices were incubated for >1 h in artificial CSF (aCSF) at room temperature (in mM): 125 NaCl, 2.5 KCl, 1 MgCl_2_, 2 CaCl_2_, 1.25 NaH_2_PO_4_, 10 glucose, and 26 NaHCO_3_, pH 7.4 and bubbled with 95% O_2_/5% CO_2_. Slices were transferred to a recording chamber and superfused (~1 ml/min) with aCSF and bubbled with 95% O_2_/5% CO_2 _at room temperature. Experiments were performed on an upright microscope (Olympus BX51WI; Olympus, Center Valley, PA, USA) under phase-contrast optics (60× objective, NA 0.9) and a 2× teleconverter. Ciliated ependymal cells were visually identified along the subventricular zone (SVZ) border lining the lateral surface of the lateral ventricles (e.g. Fig. 1A). Agonists and antagonists were bath applied, and only one exposure or experimental condition was permitted per slice. After preliminary time course experiments (see Fig. 1B), ciliary beat frequency measurements were analyzed at baseline then five min after agonist application unless otherwise indicated. Antagonists and inhibitors were always pre-applied (range 4-15 min), depending on the site of action (extracellular versus intracellular), and our prior experience using these agents in patch clamp experiments [17]; they were also present during agonist applications (for antagonist experiments only) to decrease the possibility of antagonist washout.

### Ciliary Beat Frequency Analysis

Ciliary beat frequency on lateral ventricle ependymal cells was analyzed using modifications of a previously published approach [15]. High-speed video acquisition of ciliary beat frequency was performed using a Pioneer A640-210 gm GigE camera (Basler Vision Technologies, Exton, PA, USA) with StreamPix3 software (Norpix Inc., Montreal, Quebec, Canada). One-sec videos along the ependymal wall were digitally acquired to a Dell Computer (Round Rock, TX, USA) at 200 frames per sec (fps). Files were converted to multi-TIFF stacks of 200 images and imported into ImageJ (NIH, Bethesda, MD, USA), where the stacks were re-sliced along a line placed across the ciliary tuft, thus creating pseudo-line scans. Ciliary beat frequency was calculated by measuring peak-to-peak intervals of periodicity evident in the pseudo-line scan and derived from the following equation, with each pixel representing 1/200 of a second.

Ten periods were measured for each video, representing cilia from 3-6 ependymal cells on average. Analysis was conducted blinded to experimental conditions and with randomized file names and chronology, thus decreasing potential bias. Ciliary beat frequency data from pharmacological studies are presented using the following two equations:

Comparison of our methodology to separate manual counting of ciliary beat frequency in the 1 sec video playback, as well as repeat analysis of duplicate files (with randomized names and chronology), yielded a strong correlation as determined by linear regression (r^2 ^= 0.905 and 0.95 respectively; data not shown).

### Immunocytochemistry

Immunocytochemistry was performed according to previously described protocols [17]. Briefly, animals were anesthetized with pentobarbital (50 mg/kg, IP), then fixed by transcardiac perfusion with phosphate buffered saline (PBS; 20 ml) followed by 4% paraformaldehyde (Electron Microscopy Sciences, Hatfield, PA, USA) in PBS (30 ml). The brains were removed and post-fixed in 4% paraformaldehyde in PBS (24 h, 4°C). 100 μm slices were then made using a series 1000 Vibratome. Slices were washed with 0.05 M tris base in 9% NaCl at pH 7.4 (TBS), permeabilized in TBS + 0.1% Triton X-100 (TBST), blocked in TBST + 10% normal donkey serum, and then incubated overnight with primary antibody at 4°C. Antibodies included 1:100 rabbit anti-S100β (Sigma, St. Louis, MO, USA), 1:1000 mouse anti-β-galactosidase (Sigma), 1:100 mouse anti-A_2A _(Upstate, Millipore, Billerica, MA, USA), and 1:100 rabbit anti-A_2B _(Santa Cruz Biotechnology, Santa Cruz, CA, USA). After washing (3 × 45 min in TBST), slices were incubated 2 h at room temperature in secondary antibody solution (donkey, Alexa Fluor^® ^488, 594, 633, and/or 647; Invitrogen, Carlsbad, CA, USA). After 3 × 30 minute washes in TBS they were mounted and coverslipped with Prolong Gold Antifade Reagent (Invitrogen) with or without 1:1000 DAPI (2-(4-amidinophenyl)-6-indolecarbamidine dihydrochloride; Invitrogen).

### β-galactosidase (β-gal) expression analysis in A_2B _reporter mice

β-gal expression in A_2B_^-/-^/β-gal reporter gene knock-in mice was studied according to a previously published protocol [19]. Mice were anesthetized with isoflurane, perfused with 20 ml PBS through the left heart ventricle, and perfusion fixed with 30 ml 2% paraformaldehyde in PBS. Brains were removed, cut into 2 mm coronal sections containing intact lateral ventricular walls, and stained for β-gal activity using X-gal staining solution: 5 mM K_3_Fe(CN)_6_, 5 mM K_4_Fe(CN)_6_·2 mM MgCl_2 _in PBS, with a final concentration of 1 mg/ml 5-bromo-4-chloro-3-indolyl-β-D-galactopyranoside (X-gal, American Bioanalytical; Nantick, MA, USA), then incubated at 37° for 6-12 h, rinsed in PBS, and stored in 4% paraformaldehyde. Sections were embedded in low melting point agarose (American Bioanalytical), resectioned to 100 μm, and mounted directly onto slides or used for subsequent immunocytochemistry as previously described.

### Calcium imaging

Acute mouse brain slices were loaded with the Ca^2+^-sensitive dye Fluo-4 AM (Invitrogen; 4 μM in dimethyl sulfoxide (DMSO) with 20% Pluronic F-127) using ependyma-directed applications by a Picospritzer II (1-2 psi; Parker Instrumentation, Cleveland, OH, USA). Slices were washed for a minimum of 10 min before recording. The Ca^2+ ^imaging system consisted of a confocal laser scanning microscope (Olympus) with a 60× water objective (NA 0.9) and Fluoview software (Olympus). Agonists in Ca^2+ ^imaging experiments were focally applied using a Picospritzer II (as above). Calcium data were analyzed using the Calsignal program [20].

### Genotyping

WT C57BL/6 (P2X7^+/+^), P2X7^-/-^, and A_2B_^-/-^/β-gal mice were maintained as separate, homozygous colonies. Genotyping was performed on all animals used in knockout-related experiments to survey for any potential errors in animal husbandry [17,19].

The A_2B_^-/-^/β-gal mice were previously bred onto a pure C57BL/6J background strain [21]. P2X7^-/- ^mice were previously backcrossed to C57BL/6 mice for 7 generations (Jackson Laboratories, JAX^® ^Mice Database; http://jaxmice.jax.org). We cannot exclude the possibility, however, that additional genetic variation exists between the C57BL/6 (P2X7^+/+^), P2X7^-/-^, and A_2B_^-/-^/β-gal strains.

### Reagents

Salts used for aCSF solution, as well as adenosine, αβmADP, ATP, BzATP, dipyridamole, NBMPR, phloridzin, and UTP were purchased from Sigma. CGS 21680, IB-MECA, 2'MeCCPA, MRS 1754, NECA, and PSB 603 were purchased from Tocris (Ellisville, MO, USA). A list of all drugs used in the present experiments is included in Table 1.

**Table 1:** List of drugs used

Abbreviation	Full Name	Site of Action*
αβmADP	Adenosine 5'-(α,β-methylene)diphosphate	Ecto-5'-nucleotidase inhibitor

Adenosine	9-β-D-Ribofuranosyladenine	Nonselective adenosine receptor agonist

ATP	Adenosine 5'-triphosphate	P2 purinergic agonist

BBG	Brilliant blue G	P2X_7 _receptor antagonist

BzATP	2'(3')-*O*-(4-benzoylbenzoyl)adenosine 5'-triphosphate triethylammonium salt	P2X purinergic agonist; more potent than ATP at P2X_7 _receptors

CGS-21680	4-[2-[[6-Amino-9-(*N*-ethyl-b-D-ribofuranuronamidosyl)-9*H*-purin-2-yl]amino]ethyl]benzenepropanoic acid hydrochloride	A_2A _adenosine receptor agonist

Dipyridamole	2,6-Bis(Diethanolamino)-4,8-dipiperidinopyrimido [5,4-d] pyrimidine	Equlibrative nucleoside transporter inhibitor

IB-MECA	1-Deoxy-1-[6-[[(3-iodophenyl)methyl]amino]-9*H*-purin-9-y l]-*N*-methyl-b-D-ribofuranuronamide	Selective A_3 _adenosine receptor agonist

2'MeCCPA	2-Chloro-*N*-cyclopentyl-2'-methyladenosine	Selective A_1 _adenosine receptor agonist

MRS 1754	*N*-(4-cyanophenyl)-2-[4-(2,3,6,7-tetrahydro-2,6-dioxo-1,3-dipropyl-1*H*-purin-8-yl)phenoxy]-acetamide	Selective adenosine A_2B _receptor antagonist

NBMPR	S-(4-Nitrobenzyl)-6-thioinosine	Equilibrative nucleoside transporter 1 inhibitor

NECA	5'-*N*-ethylcarboxamidoadenosine	Nonselective adenosine receptor agonist.

Phloridzin	Phloretin 2'-β-D-glucopyranoside	Concentrative nucleoside transporter inhibitor

PSB 603	8-[4-[4-(4-Chlorophenzyl)piperazide-1-sulfonyl)phenyl]]-1-propylxanthine	Selective adenosine A_2B _receptor antagonist

Suramin	8,8'-[Carbonylbis[imino-3,1-phenylenecarbonylimino(4-me thyl-3,1-phenylene)carbonylimino]]bis-1,3,5-naphthalene trisulfonic acid hexasodium salt	P2 antagonist (with broad subtype selectivity)

UTP	Uridine 5'-triphosphate	P2Y_2_/P2Y_4 _receptor agonist

* Selectivity depends on the concentration tested. Non-purinergic activities may also be present.

### Statistics

Data were analyzed and presented in SigmaPlot 8.0 (SPSS, Chicago, IL, USA). Statistical significance was determined using the Student's t-test (*P *< 0.05). Data are presented as mean ± standard error of the mean (SEM) unless otherwise indicated. Reported *n *values refer to the number of slices tested (with each slice including 10 ciliary beat frequency measurements; see above).

## Results

### Purinergic enhancement of ciliary beat frequency is present in P2X7^+/+ ^and P2X7^-/- ^mice and is induced by non-selective adenosine receptor agonist

Given our prior demonstration of ciliary (and somatic) localization of P2X_7 _receptors on lateral ventricle ependymal cells [17], we first sought to determine whether BzATP (a commonly used P2X_7 _agonist) was also able to induce changes in ciliary beat frequency. Ciliated ependymal cells were visualized in horizontal mouse brain slices using high-speed DIC microscopy (Fig. 1A; see *Methods *for ciliary beat frequency calculations). Average baseline ciliary beat frequency was 11.4 ± 0.2 Hz (n = 160) in wild-type mice. While 300 μM BzATP (Fig. 1B) was able to increase ciliary beat frequency in C57BL/6 wild-type (P2X_7_^+/+^) mice, a similar increase was also observed in *P2X*_7_^-/- ^animals. Fig. 1C shows the % increase after a 5 min application of 300 μM BzATP in P2X_7_^+/+ ^mice (58.9 ± 3.4%) and in P2X_7_^-/- ^mice (58.6 ± 4.1%). These responses were not significantly different.

Preincubation with 100 nM brilliant blue G (BBG; a P2X_7 _antagonist), however, did result in a partial inhibition of BzATP-induced beat frequency enhancement in P2X_7_^+/+ ^animals (Fig. 1C; 46.1 ± 4.4%). This suggests that P2X_7 _may contribute only a minor component to BzATP-induced enhancement in wild-type mice. A significant decrease in BzATP-induced enhancement, *P *< 0.05, however, was also observed when the ecto-5'-nucleotidase inhibitor αβmADP (50 μM, [22]) was present in the bath solution for both P2X_7_^+/+ ^mice (35.1 ± 3.8%) and P2X_7_^-/- ^mice (27.2 ± 3.2%, Fig. 1C) thus providing evidence that enhancement may be dependent on a metabolic breakdown product of BzATP.

To determine if either P2Y receptors or adenosine receptors are involved in ciliary beat frequency enhancement, experiments were performed using ATP, UTP, and a P2Y antagonist suramin (Fig. 1D), as well as adenosine and the non-selective adenosine receptor agonist 5'-*N*-ethylcarboxamidoadenosine (NECA) (Fig. 1E). While beat frequency increase was observed during 100 μM ATP applications (47.1 ± 6.6%), pre-incubation with the commonly used P2Y receptor antagonist suramin (200 μM) did not reduce the ATP-induced increase in frequency (Fig. 1D; 52.6 ± 4.8%). Furthermore, application of the P2Y_2_/P2Y_4 _agonist UTP (Fig. 1D, 100 μM, 4.4 ± 3.2%) did not induce a significant change in baseline frequency, arguing against a role for these receptors in the ATP-induced effect. Dose-response experiments (Fig. 1E) showed that beat frequency increased with adenosine (1 μM, 18.7 ± 3.7%, n = 5; 10 μM, 35.1 ± 5.6%, n = 5; 30 μM, 67.9 ± 5.1%, n = 5; 60 μM, 56.5 ± 5.3%, n = 4) and with NECA (100 nM, 9.4 ± 2.6%, n = 5; 1 μM, 41.9 ± 3.4%, n = 8; 10 μM, 45.6 ± 5.1%, n = 4), and that NECA had a greater potency than adenosine.

### A_2B _receptors mediate adenosine and NECA-induced ciliary beat frequency enhancement

**Figure 2:**
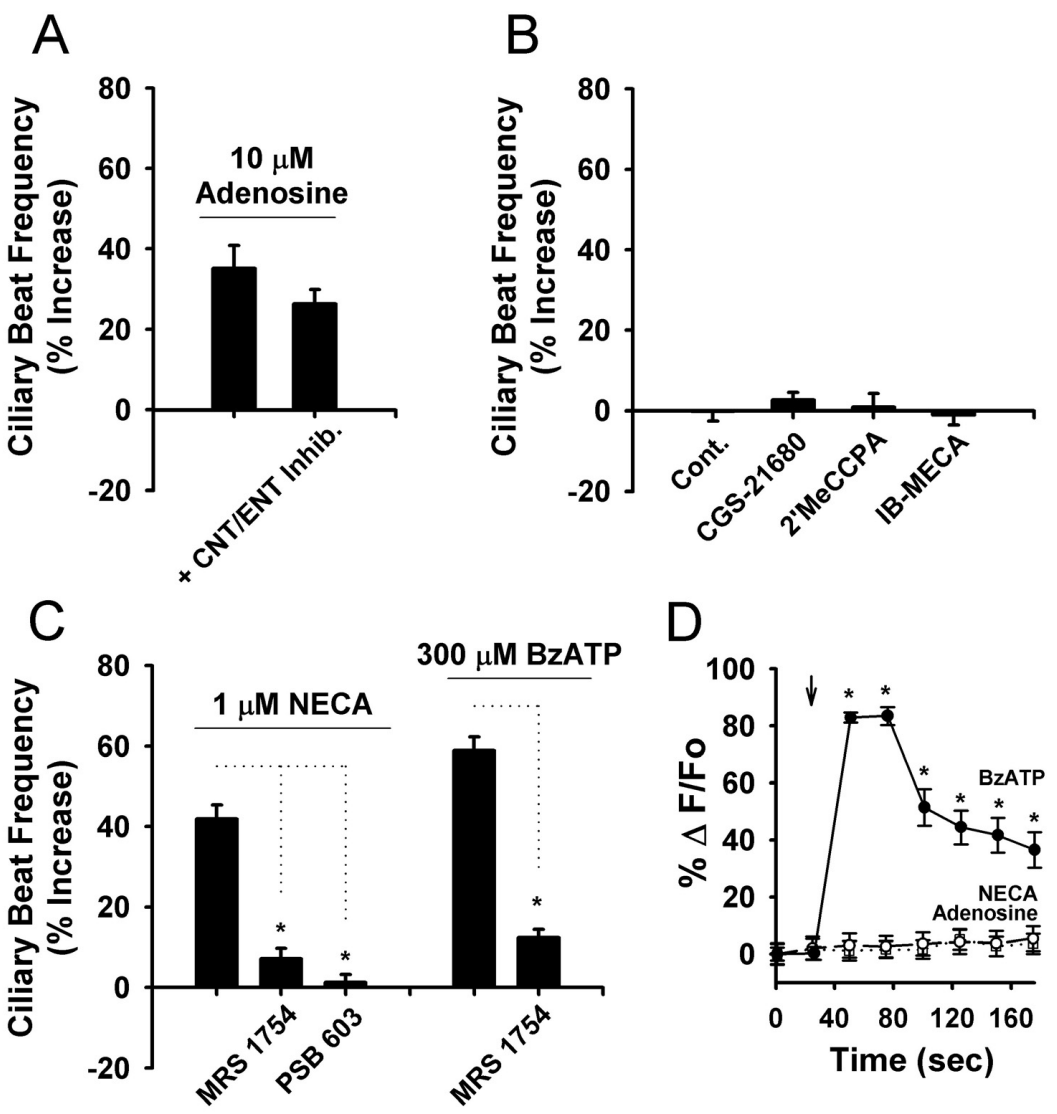
**Adenosine-mediated enhancement of ciliary beat frequency is due to A_2B _receptor activation**. (*A*) Histogram showing that ciliary beat frequency enhancement was not eliminated by pre-incubation with a cocktail of CNT and ENT inhibitors (1 mM phloridzin, 100 μM dipyridamole, 10 μM NBMPR; n = 5). (*B*) Summary histogram showing that ciliary beat frequency did not increase in response to a no drug control (Cont., n = 10), nor selective concentrations of the A_2A _agonist CGS 21680 (100 nM, n = 5), the A_1 _agonist 2'MeCCPA (100 nM, n = 5), nor the A_3 _agonist IB-MECA (100 nM, n = 5). There was no significant difference between the no drug control and CGS 21680, 2'MeCCPA, or IB-MECA. (*C*) Histogram demonstrating that the response to 1 μM NECA was significantly reduced by the A_2B _antagonist MRS 1754 (100 nM, n = 9) and eliminated by the A_2B _antagonist PSB 603 (1 μM, n = 10). Response to 300 μM BzATP was also reduced by MRS 1754 (100 nM, n = 8). (*D*) [Ca^2+^]_i _was increased by 1 min focal application (↓) of 300 μM BzATP ([Black circle], n = 1 application/10 regions of interest), but not by 3 min applications of 1 μM NECA (○, n = 3 applications/30 regions of interest) or 30 μM adenosine, (□, n = 3 applications/30 regions of interest): note: ○ and □ symbols largely overlap. The percent change in fluorescence signal divided by baseline mean fluorescence intensity is shown in the Y-axis (%ΔF/F_0_). *: *P *< 0.05 for all panels, data are means ± SEM. The *n *value indicates the number of slices tested.

A cocktail of inhibitors for equilibrative (ENT) and concentrative (CNT) nucleoside transporters (1 mM phloridzin, 100 μM dipyridamole, 10 μM NBMPR; [23-25]) was not able to significantly reduce adenosine-induced enhancement of beat frequency (Fig 2A; 26.3 ± 3.6%, *P *= 0.19), suggesting that neither influx nor efflux of adenosine analogs is responsible for adenosine's effect on beat frequency. Furthermore, selective (100 nM) concentrations of the adenosine receptor agonists CGS 21680 (A_2A_; 2.7 ± 1.2%,), 2'MeCCPA (A_1_; 0.9 ± 3.4%,), or IB-MECA (A_3_; -1.0 ± 2.5%) did not reveal any enhancement of beat frequency (Fig. 2B) when compared to a no drug control (-0.1 ± 2.4%). Loss of subtype selectivity precluded the use of higher concentrations of these agonists for receptor identification. As a selective A_2B _receptor agonist was not commercially available [26], we tested two selective A_2B _receptor antagonists for their ability to block the NECA-induced enhancement of frequency. As shown in Fig. 2C, MRS 1754 (100 nM; 7.1 ± 2.7%) and PSB 603 (1 μM; 1.3 ± 2.0%) significantly blocked the increase in frequency induced by 1 μM NECA. Furthermore, enhancement of beat frequency by 300 μM BzATP was also significantly reduced by MRS 1754 application (Fig. 2C; 12.4 ± 2.0%), demonstrating that the previously observed BzATP response is primarily due to A_2B _receptor activation. A residual P2X_7_-mediated component cannot, however, be excluded.

In Ca^2+ ^imaging experiments, focal applications of 1 μM NECA or 30 μM adenosine onto ependymal cells did not induce a change in [Ca^2+^]_i_, thus suggesting that A_2B_-mediated enhancement of beat frequency is not Ca^2+^-mediated (Fig. 2D). Separate beat frequency experiments demonstrated that 100 μM adenosine induces ciliary beat frequency enhancement in EGTA (ethylene glycol-bis(2-aminoethylether)-N, N, N', N'-tetraacetic acid)-buffered Ca^2+^-free external solution (46.4 ± 8.2%, n = 2 slices, *P *< 0.05, data not shown), thereby supporting the conclusion that A_2B_-mediated enhancement of beat frequency is not Ca^2+^-mediated. Finally, as a positive control (and consistent with prior observations from our laboratory [17]), 300 μM BzATP-induces a dramatic increase in [Ca^2+^]_i _(Fig. 2D).

### Histochemical and functional evidence for A_2B _expression: immunocytochemistry and A_2B_^-/-^/β-gal reporter gene knock-in mice

**Figure 3:**
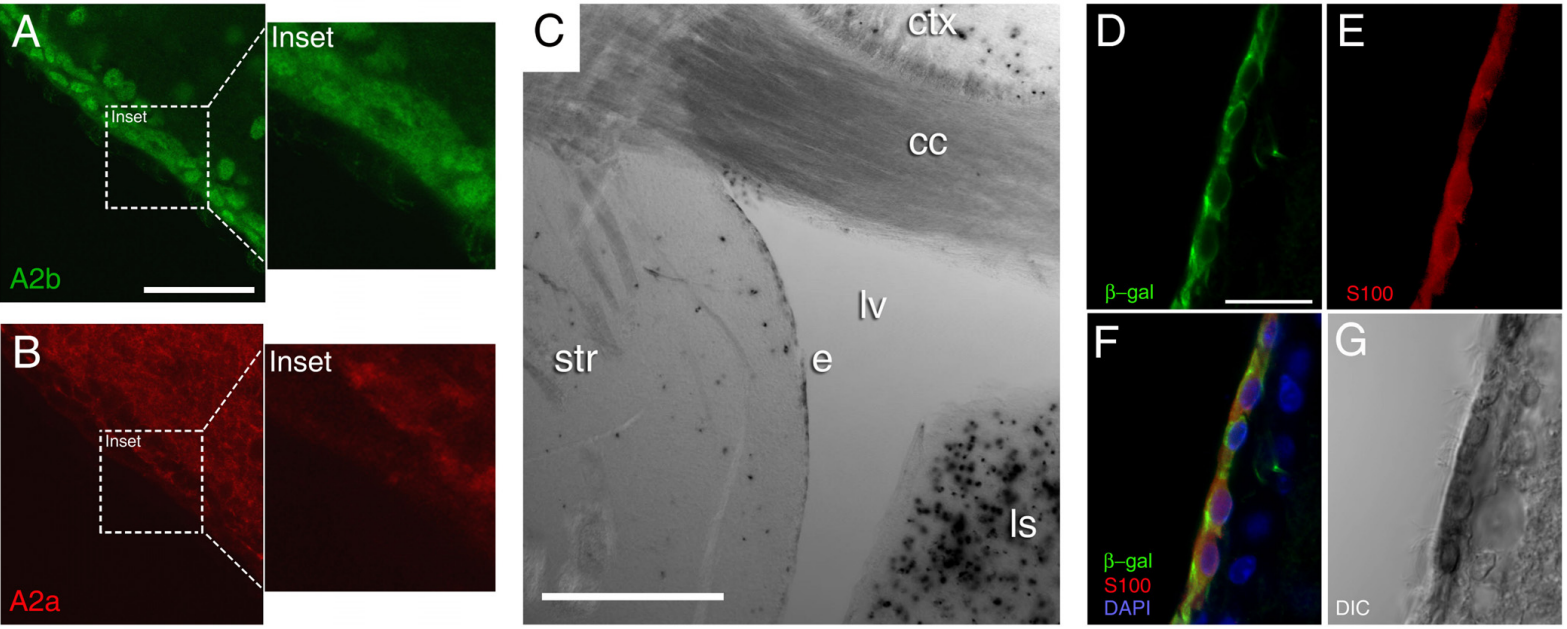
**Ependymal localization of A_2B_: evidence from immunocytochemistry and X-gal staining**. (*A*) Cytoplasmic A_2B_-immunoreactivity was evident in ependymal cells (see inset) of wild type mice, although non-specific nuclear labeling was also evident throughout the brain and confounds interpretation of ependymal immunoreactivity. (*B*) No labeling of ependymal cells was observed using an antibody to A_2A _receptors in wild type mice, although strong immunoreactivity was evident in the striatum and in a scattered distribution along the SVZ. (*C*) DIC image from an A_2B_^-/-^/β-gal reporter gene knock-in mouse showing darkening of cells due to X-gal precipitate in regions surrounding the ependymal layer (*e*). Strong X-gal labeling was observed in the lateral septal nucleus (*ls*), while scattered labeling was observed in the striatum (*st*) and cortex (*ctx*) but not in the corpus callosum (*cc*). The septum mechanically separated from the corpus callosum during the staining procedure, thus obliterating the dorso-medial boundary of the lateral ventricle (*lv*) in this slice. (Bar = 500 μm). (*D-F*) Immunocytochemistry from an A_2B_^-/-^/β-gal reporter gene knock-in mouse demonstrating that β-galactosidase (*D*; green, Bar = 25 μm) and S100β (*E*, red) are co-localized in ependymal cells (*F*). Nuclei are stained with DAPI (blue). (*G*) Corresponding DIC image with darkening of the ependyma due to X-gal precipitate.

We next sought to confirm A_2B _expression by ependymal cells using immunocytochemical methods. While distinct A_2B _immunoreactivity was observed in ependymal cells (Fig. 3A), non-selective nuclear staining was also observed throughout the central nervous system (CNS) and therefore precluded definitive interpretation. Two additional A_2B _antibodies did not show any CNS labeling (data not shown). A_2A _immunoreactivity was evident in the striatum and in a scattered distribution along the SVZ but not in ependymal cells (Fig. 3B). An alternative approach was therefore used as a verification of the presence of A_2B _receptors.

Previously characterized A_2B_^-/-^/β-gal reporter gene knock-in mice [19] were surveyed for A_2B _gene promoter-driven expression of β-galactosidase in ependymal cells along the lateral ventricle. Strong X-gal reaction product was observed in the lateral septal nucleus, and clear intracellular labeling was also visible in ependymal cells and scattered throughout the cortex and striatum (Fig. 3C). This pattern was observed in A_2B_^-/-^/β-gal mice but not in wild-type controls. β-gal immunoreactivity was also observed in the A_2B_^-/-^/β-gal mice in S100β-positive ependymal cells (Fig. 3D-F), providing an additional layer of evidence for A_2B _expression by ependyma. Ependymal X-gal reaction product is also visible in the corresponding Fig. 3G.

**Figure 4:**
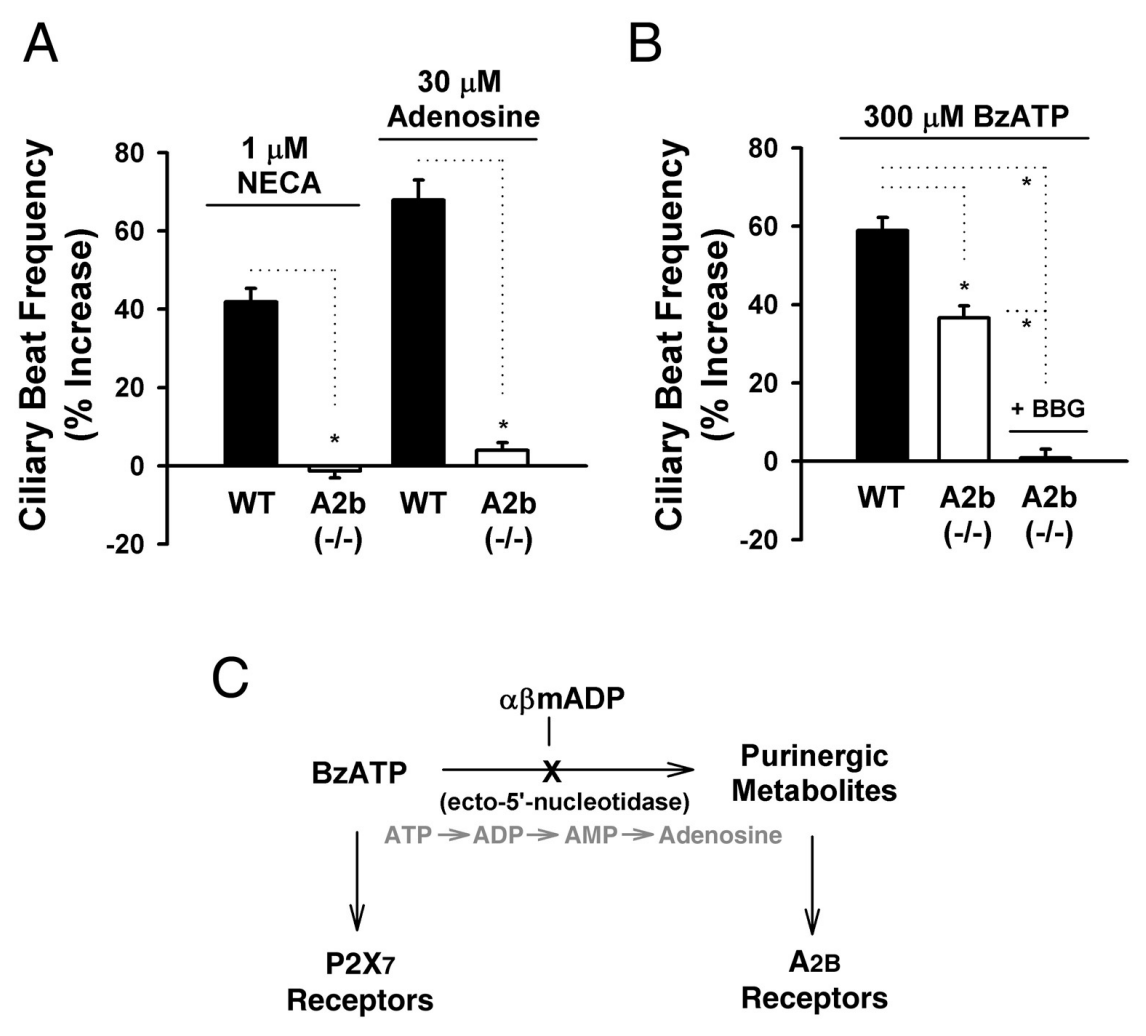
**Ciliary beat frequency analysis in A_2B_^-/-^/β-gal reporter gene knock-in mice**. (*A*) Histogram showing the absence of ciliary beat frequency enhancement due to 1 μM NECA (n = 10) and 30 μM adenosine (n = 10) in the A_2B_^-/- ^mice. (*B*) Enhancement due to 300 μM BzATP application was reduced in the A_2B_^-/- ^mice (n = 8) versus wild-type P2X_7_^+/+ ^mice (n = 5). BzATP-induced enhancement was eliminated in the A_2B_^-/- ^mice after pre-incubation of the slices with 100 nM BBG (n = 8). *: *P *< 0.05 for all panels, data are means ± SEM. The *n *value indicates the number of slices tested. (*C*) Summary diagram showing enzymatic breakdown of BzATP and subsequent receptor activation. ATP (an endogenous signaling molecule analogous to BzATP) is shown in grey.

Functional evidence for A_2B _expression by ependymal cells was also observed in the A_2B_^-/-^/β-gal mice (Fig. 4). Neither NECA (1 μM; -1.3 ± 1.8%) nor adenosine (30 μM; 4.0 ± 1.9%) was capable of increasing ciliary beat frequency in the A_2B_^-/-^/β-gal mice (Fig. 4A). BzATP, however, induces a significant (Fig. 4B), albeit smaller, enhancement of beat frequency in A_2B_^-/-^/β-gal mice (36.6 ± 3.0%) versus wild-type controls (58.9 ± 3.4%, data also in Fig. 1C). The increase induced by BzATP in the A_2B_^-/-^/β-gal mice is completely blocked by pre-incubation with the P2X_7 _antagonist BBG (Fig. 4B; 0.8 ± 2.2%), thus providing additional evidence for a residual P2X_7_-mediated enhancement in these animals. A summary diagram is presented in Fig. 4C.

## Discussion

The present experiments demonstrated that activation of the adenosine A_2B _receptor enhanced ciliary beat frequency in mouse lateral ventricle ependymal cells - a conclusion supported by pharmacological experiments using selective adenosine receptor agonists and antagonists, as well as experiments using the A_2B_^-/-^/β-gal mice. The fact that BzATP application onto mouse brain slices can lead to activation of a non-P2X_7_-mediated pathway is not surprising. For example, prior studies in the hippocampus have demonstrated that BzATP can induce non-P2X_7_-mediated effects through the action of ecto-nucleotidases, nucleoside transporters, and subsequent adenosine receptor activation [27]. Ependymal cells have been shown to express ecto-nucleotide pryrophasphatase/phosphodiesterase 1 (NPP1) and ecto-5'-nucleotidase [28,29], and the decrease in BzATP-mediated effects after pre-incubation with αβmADP (an ecto-5'-nucleotidase inhibitor; Fig. 1C) suggests that ciliary beat frequency enhancement is largely dependent on a metabolic breakdown product rather than BzATP itself.

It should be noted that BBG was used as the sole P2X_7 _antagonist in these studies, as the more commonly used adenosine 5'triphosphate-2',3'-dialdehyde (oATP) induced toxicity in prior experiments (unpublished observations) and KN-62 has demonstrated a weaker activity at mouse versus human P2X_7 _receptors [30]. Our recent whole-cell patch clamp experiments, however, showed clear antagonism of ependymal cell BzATP-induced currents by low concentrations of BBG [17]. Furthermore, no additional P2X receptor subtypes were detected during patch clamp recordings of P2X_7_^-/- ^mice [17]. While data in Fig. 1C, Fig. 2C, and Fig. 4B argue that a minor P2X_7_-mediated component to BzATP-induced ciliary beat frequency enhancement is present, it is most easily observed in the absence of the adenosine A_2B _receptor (Fig. 4B).

The lack of ciliary beat frequency enhancement with 100 μM UTP (Fig. 1D), and the absence of a suramin-mediated antagonism of ATP-induced changes in beat frequency (Fig. 1D), strongly argue against a P2Y-mediated modulation of frequency in the present experiments. These data do not altogether eliminate the possibility, however, that another subtype of P2Y-receptor may play a role in beat frequency modulation. A more extensive pharmacological analysis (with inclusion of appropriate ecto-nucleotidase inhibitors to prevent breakdown of purinergic drugs into adenosine receptor agonists) is clearly desirable and should be the focus of future investigation.

Interestingly, ATP has previously been shown to decrease ciliary beat frequency in rat 4^th ^ventricle ependymal cells [14]. It is reasonable to assume that species and region-specific differences may exist in ependymal cell response to ATP, which is obviously dependent on the subtypes of purinergic receptors expressed. For example, in our mouse lateral ventricle ependymal cells, [Ca^2+^]_i _increases rapidly after BzATP application (Fig. 2D and [17]); this is in sharp contrast to ATP's lack of [Ca^2+^]_i _effect in the previously mentioned rat experiments [14]. Other proteins expressed by ependyma during development - such as glial fibrillary acidic protein (GFAP) and vimentin - vary markedly between species, developmental stage, and location along the ventricular system [31]. Future work on anatomic as well as species-specific differences in ependymal cell ciliary beat frequency regulation is clearly warranted.

A_2B _can be coupled to multiple G-protein cascades, including the adenylate cyclase (Gs; cAMP) pathway and the phospholipase C (Gq11) signaling pathways [32-35]. Furthermore, activation of the phospholipase C - mediated pathway can lead to [Ca^2+^]_i _increases after A_2B _activation [32]. In the present experiments, however, neither NECA (1 μM) nor adenosine (30 μM) were able to induce [Ca^2+^]_i _increases in ependymal cells (Fig. 2D), arguing against a Ca^2+^-mediated mechanism for A_2B_-induced enhancement of ciliary beat frequency. While additional pathways involved in A_2B_-mediated signaling were not explored in the present experiments, a complete understanding of these pathways may prove critical for determining the importance of receptor signaling cascades in CSF dynamics. For example, a recent study by Mönkkönen *et al*. (2007) has demonstrated that knockdown of G_αi2 _can lead to ciliary stasis and ventricular dilation [11].

Nucleotide signaling and purinergic receptor expression in the developing brain has been the subject of intense investigation (for review, see [36]). For example, the developmental precursors of ependyma - radial glia [37] - can propagate ATP-mediated Ca^2+ ^waves that are dependent on P2Y_1 _receptor expression [38]. Immature ependyma are born between embryonic days E14 and E16 in the mouse, although cell maturation and cilia formation typically occur during the first postnatal week [37]. Little is known regarding the functional role of purinergic receptors on these cells during this time. It should also be noted that neuroblast migration from the SVZ to the rostral migratory stream depends on the normal flow of CSF, and ciliary motility is required for maintaining a diffusional gradient of inhibitory guidance molecules in the CSF [39]. Whether receptor-mediated changes in ciliary beat frequency play a role in this phenomenon is not known. Purinergic receptor expression on CSF secreting cells of the choroid plexus has also been the subject of recent investigations [40,41].

Additional questions clearly remain to be answered. Is the source of endogenous ATP or adenosine autocrine or paracrine? Does ciliary beat frequency correlate with the metabolic requirements in the CNS, and might ciliary beat frequency dysregulation be associated with hydrocephalus? While answers to these questions are beyond the scope of the present experiments, much remains to be learned about the role of purinergic receptors and ciliary beat frequency in cerebral fluid dynamics.

## Conclusion

While abnormal ciliary structure and function has been associated with hydrocephalus in several experimental models, the signaling mechanisms responsible for the normal regulation of ependymal cell ciliary beat frequency are not well understood. The present experiments demonstrate that activation of the adenosine A_2B _receptor enhances ciliary beat frequency in lateral ventricle ependymal cells. A residual contribution of purinergic P2X_7 _receptors to frequency regulation is also supported. Purinergic modulation of ependymal cell beat frequency may play an important role in maintaining normal fluid balance in the CNS. Future experiments should focus on understanding whether purinergic dysregulation contributes to pathologic conditions such as hydrocephalus.

## Abbreviations

**aCSF**: artificial cerebrospinal fluid; **αβmADP**: adenosine 5'-(α,β-methylene)diphosphate; **ATP**: adenosine 5'-triphosphate; **BBG**: brilliant blue G; **β-gal**: β-galactosidase; **BzATP**: 2'(3')-*O*-(4-benzoylbenzoyl)adenosine 5'-triphosphate triethylammonium salt; **cAMP**: adenosine 3',5'-cyclic monophosphate; **CGS-21680**: 4-[2-[[6-Amino-9-(*N*-ethyl-b-D-ribofuranuronamidosyl)-9*H*-purin-2-yl]amino]ethyl]benzenepropanoic acid hydrochloride; **CNT**: concentrative nucleoside transporter; **DAPI**: (2-(4-Amidinophenyl)-6-indolecarbamidine dihydrochloride); **DIC**: differential interference contrast; **DMSO**: dimethyl sulfoxide; **ENT**: equilibrative nucleoside transporter; **IB-MECA**: 1-deoxy-1-[6-[[(3-iodophenyl)methyl]amino]-9*H*-purin-9-y l]-*N*-methyl-b-D-ribofuranuronamide; **2'MeCCPA**: 2-chloro-*N*-cyclopentyl-2'-methyladenosine; **MRS 1754**: *N*-(4-cyanophenyl)-2-[4-(2,3,6,7-tetrahydro-2,6-dioxo-1,3-dipropyl-1*H*-purin-8-yl)phenoxy]-acetamide; **NBMPR**: S-(4-Nitrobenzyl)-6-thioinosine; **NECA**: 5'-*N*-ethylcarboxamidoadenosine; **NPP1**: ecto-nucleotide pryrophasphatase/phosphodiesterase 1; **oATP**: adenosine 5'triphosphate-2',3'-dialdehyde; **PBS**: phosphate buffered saline; **PSB 603**: 8-[4-[4-(4-chlorophenzyl)piperazide-1-sulfonyl)phenyl]]-1-propylxanthine; **SVZ**: subventricular zone; **TBS**: Tris buffered saline; **TBST**: TBS + 0.1% Triton X-100; **UTP: **uridine 5'-triphosphate.

## Competing interests

The authors declare that they have no competing interests.

## Authors' contributions

JRG. and AB designed research; JRG performed ciliary beat frequency, immunocytochemistry, and calcium imaging experiments at Yale University; KR and DY developed the A2b KO mouse at Boston University; DY performed X-gal staining; JRG analyzed data and wrote the manuscript. All authors have read and approved the final version of the manuscript.
